# A Citrus and Pomegranate Complex Reduces Methylglyoxal in Healthy Elderly Subjects: Secondary Analysis of a Double-Blind Randomized Cross-Over Clinical Trial

**DOI:** 10.3390/ijms241713168

**Published:** 2023-08-24

**Authors:** Katarzyna Bednarska, Izabela Fecka, Jean L. J. M. Scheijen, Sanne Ahles, Philippe Vangrieken, Casper G. Schalkwijk

**Affiliations:** 1 Department of Pharmacognosy, Faculty of Pharmacy, Wroclaw Medical University, Borowska 211, 50-556 Wroclaw, Poland; 2 The Committee on Therapeutics and Pharmaceutical Sciences, The Polish Academy of Sciences, Pl. Defilad 1, 00-901 Warsaw, Poland; 3 Department of Internal Medicine, Maastricht University Medical Center+, 6229 ER Maastricht, The Netherlands; j.scheijen@maastrichtuniversity.nl (J.L.J.M.S.); p.vangrieken@maastrichtuniversity.nl (P.V.); c.schalkwijk@maastrichtuniversity.nl (C.G.S.); 4 CARIM School for Cardiovascular Diseases, Faculty of Health, Medicine and Life Sciences, Maastricht University, 6229 ER Maastricht, The Netherlands; 5 Department of Nutrition and Movement Sciences, School of Nutrition and Translational Research in Metabolism (NUTRIM), Maastricht University, 6229 ER Maastricht, The Netherlands; s.ahles@maastrichtuniversity.nl; 6 BioActor BV, 6229 GS Maastricht, The Netherlands

**Keywords:** hesperidin, punicalagin, α-dicarbonyls, methylglyoxal, glyoxal, 3-deoxyglucosone, *Punica* × *granatum*, *Citrus* × *sinensis*

## Abstract

Reactive α-dicarbonyls (α-DCs), such as methylglyoxal (MGO), glyoxal (GO), and 3-deoxyglucosone (3-DG), are potent precursors in the formation of advanced glycation end products (AGEs). In particular, MGO and MGO-derived AGEs are thought to be involved in the development of vascular complications in diabetes. Experimental studies showed that citrus and pomegranate polyphenols can scavenge α-DCs. Therefore, the aim of this study was to evaluate the effect of a citrus and pomegranate complex (CPC) on the α-DCs plasma levels in a double-blind, placebo-controlled cross-over trial, where thirty-six elderly subjects were enrolled. They received either 500 mg of *Citrus sinensis* peel extract and 200 mg of *Punica granatum* concentrate in CPC capsules or placebo capsules for 4 weeks, with a 4-week washout period in between. For the determination of α-DCs concentrations, liquid chromatography tandem mass spectrometry was used. Following four weeks of CPC supplementation, plasma levels of MGO decreased by 9.8% (−18.7 nmol/L; 95% CI: −36.7, −0.7 nmol/L; *p* = 0.042). Our findings suggest that CPC supplementation may represent a promising strategy for mitigating the conditions associated with MGO involvement. This study was registered on clinicaltrials.gov as NCT03781999.

## 1. Introduction

Despite substantial advances in diabetes treatment, vascular complications remain a significant clinical concern and are the primary cause of mortality among diabetes patients [1]. Several different biochemical pathways are involved in diabetes-induced endothelial damage and vascular complications, but an elevated level of systemic α-dicarbonyl compounds (α-DCs) is considered one of the key factors in the development of vascular complications [2]. The α-DCs are a subclass within the large group of compounds collectively referred to as reactive carbonyl species (RCS). These compounds feature two carbonyl groups (>C=O) on the same molecule, typically at consecutive carbon atoms, leading to the ‘alpha’ or ‘α’ designation. Within these compounds, the C-1 and C-2 carbons bond directly to oxygen, forming a keto-aldehyde or dialdehyde structure with two neighboring carbonyl groups. The most investigated α-dicarbonyl compounds include methylglyoxal (MGO, CH3-C(=O)-CH(=O)), glyoxal (GO, O=CH-CH(=O)), and 3-deoxyglucosone (3-DG, OHCH2-(CHOH)2-CH2-C(=O)-CH(=O)) [3]. The state of increased α-DCs is termed carbonyl stress. Among them, MGO is the most reactive and plays a significant role as a precursor in the non-enzymatic glycation process, ultimately resulting in advanced glycation end-product (AGE) formation [4]. The process of non-enzymatic glycation involves the excessive nucleophilic addition of the carbonyl groups of reducing sugars or α-DCs to the amino and guanidino groups of various biomacromolecules, such as peptides, proteins, lipoproteins, and nucleic acids. MGO is produced predominantly through the non-enzymatic degradation of triose phosphates, glyceraldehyde-3-phosphate, and dihydroxyacetone phosphate, as a metabolic side product of glucose metabolism during glycolysis [5]. Additionally, it can be formed through the non-enzymatic oxidation of glucose and fructose and early glycation products (Amadori products) [3]. Thus, excessive consumption of simple sugars may contribute to increased MGO and other α-DCs concentrations in tissues and plasma. A presumably similar mechanism of MGO formation occurs in individuals with poorly controlled glycemia, in whom glucose concentrations increase significantly over a long period [6].

A natural consequence of the modification of biomacromolecules by MGO is the loss of their original spatial structure. This results in a loss of physiological function, leading to various cellular disorders [7,8]. The major MGO-derived AGE, *N*^δ^-(5-hydro-5-methyl-4-imidazolone-2-yl)ornithine (MG-H1), produced in the reaction of MGO with arginine, has been positively correlated with the occurrence of vascular endothelial damage [9,10]. Another AGE, formed analogously during the reaction of MGO with lysine *N*^ε^-(1-carboxyethyl)lysine, known as CEL, was found to correlate positively with insulin resistance in diabetic patients [11]. Moreover, AGEs accumulate in the extracellular space in tissues and organs. Their role in the development of cardiometabolic and neurodegenerative diseases has been demonstrated in numerous studies [12,13,14,15]. The glyoxalase system, comprising glyoxalase I (Glo1) and glyoxalase II (Glo2), primarily detoxifies MGO in the human body, maintaining relatively low intracellular MGO levels under normal physiological conditions [16,17]. However, when the glyoxalase system is overwhelmed or not functioning, factors, such as permanently elevated glucose levels or deficiencies in Glo1/Glo2, can lead to the accumulation of MGO in cells and tissues [18].

Excessive MGO formation can further increase the production of reactive oxygen species and nitrogen species and consequently induce oxidative stress, which further contributes to the development and progression of vascular complications in diabetes [19]. MGO-derived AGEs, through the RAGE receptor and nuclear factor NF-κB, trigger an increase in the secretion of pro-inflammatory cytokines, interleukins 6 and 8, and tumor necrosis factor and contribute to the enhanced production of superoxide anion, peroxynitrite, and hydrogen peroxide [20,21]. Oxidative stress and inflammation are suspected to play key roles in MGO-induced vascular endothelial damage [22].

Preventing diabetes vascular complications necessitates a multidirectional therapeutic approach, as simply targeting risk factors, like glycemic control and a healthy lifestyle, may not be sufficient. Diabetes is a complex metabolic disorder, and the pathogenesis of its vascular complications involves multiple interlinked biochemical, molecular, and physiological processes and, therefore, requires multidirectional therapy. Lowering systemic levels of MGO, GO, and 3-DG may delay the formation of AGEs, particularly crucial in patients experiencing pathophysiological increases in α-DCs (dicarbonyl stress) due to diabetes.

To date, several agents have been studied and proposed to be useful as an anti-MGO therapy. Among them are experimental compounds, such as aminoguanidine [23] and alagebrium [24]; registered drugs, such as metformin [25], thiazolidinediones [26], angiotensin-converting enzyme inhibitors [27], angiotensin receptor blockers [28,29], and statins [30]; and supplements, such as L-carnosine [31], pyridoxamine [32], or thiamine (vitamin B1) [33]. However, both aminoguanidine and alagebrium were excluded from further phases of clinical trials due to side effects resulting in safety concerns. For registered drugs and supplements, excluding metformin [25], the absence of clear evidence of efficacy in human clinical trials has led to the discontinuation of attempts to employ them in pharmacotherapy [34].

Plant-derived dietary supplements and nutraceuticals are becoming increasingly popular all over the world as a result of growing customer awareness regarding their potential health benefits [35]. It is well established that some plant polyphenols like flavonoids have beneficial effects on general cardiometabolic health and may modulate the risk of the development of cardiovascular and metabolic diseases, including diabetes and its vascular complications [36,37]. The literature provides studies on the phytochemical composition of *Citrus sinensis* and *Punica granatum* and confirms that both plants are abundant in bioactive polyphenolic components, mainly flavonoids in the case of sweet orange and ellagitannins in the case of pomegranate fruit [38,39].

Several experimental in vitro studies have proved that citrus and pomegranate polyphenols can scavenge reactive α-dicarbonyls and prevent the formation of AGEs [40,41,42,43,44,45]. Hesperidin, the primary flavonoid glycoside in sweet orange extract, forms adducts with MGO and GO, potentially reducing their concentrations in the system. Hesperidin aglycone, hesperetin, also has MGO and GO trapping ability and is a known inducer of Glo1 [40,46]. Pomegranate fruit extract’s principal polyphenolic compounds, punicalagin and ellagic acid, exhibit potent antioxidant activity. Some in vitro experimental studies report that ellagic acid and urolithin A, a gut microbiota metabolite of ellagitannins and ellagic acid, inhibit non-enzymatic glycation by scavenging MGO [43].

Despite experimental evidence, controlled human studies on the effects of orange and pomegranate actives on α-DCs levels are lacking. Therefore, this post hoc analysis of a double-blind, randomized, cross-over clinical trial in apparently healthy elderly participants examines the impact of a combination of citrus extract and pomegranate concentrate (CPC) on α-DCs levels. The results may shed light on potential therapeutic approaches to inhibit hyperglycemia-induced vascular endothelial damage.

## 2. Results and Discussion

A total of 42 healthy participants were initially screened for eligibility in the study, out of which 37 volunteers were enrolled, and 36 completed both the placebo and treatment phases, with no dropouts during the follow-up period. The final study population comprised 27 females and 9 males. The average age ± SD of the study population at the start of the study was 66 ± 4 y, and the average BMI was 25.3 ± 0.3 kg/m^2^. The basic baseline characteristics of the study subjects and α-dicarbonyl concentrations at the beginning of the study are summarized in Table 1.

The study population, despite being elderly, was deemed apparently healthy. The baseline mean MGO concentration for the participants was 190.1 nmol/L. This falls within the accepted physiological range of 60–250 nmol/L, as measured using liquid chromatography–tandem mass spectrometry [47,48]. The baseline GO concentration in the study was 117.7 nmol/L, which is notably lower compared to concentrations observed in healthy non-diabetic populations, typically ranging from 330 to 1150 nmol/L [49,50]. Meanwhile, the average baseline concentration of 3-DG was 569.2 nmol/L, aligning with the reported range of 160–1046 nmol/L for healthy individuals [49].

The 4-week treatment with CPC resulted in a significant decrease in plasma MGO concentrations compared with the placebo treatment, showing a reduction of 18.7 nmol/L (9.8% reduction from baseline). However, the decrease in GO and 3-DG concentrations with CPC treatment was not statistically significant, with reductions of 7.8 nmol/L (6.6% reduction from baseline) and 16.6 nmol/L (2.9% reduction from baseline), respectively. The total treatment effect is summarized in Table 2.

**Figure 1:**
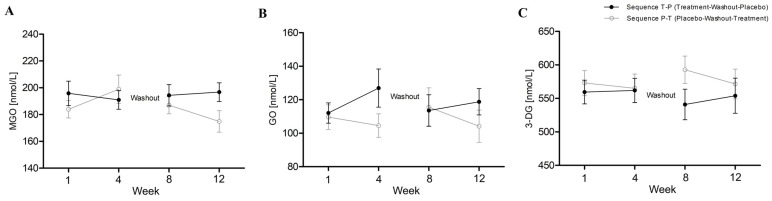
Changes in α-dicarbonyls concentrations during the study expressed in nmol/L unit. The T-P sequence represents a group of participants who first received the CPC treatment and then, after a washout period, were given a placebo. The P-T sequence refers to those participants who initially received the placebo and then, following a washout period, were administered the CPC. Data are presented as actual mean ± SEM (standard error of measurement). Fasting plasma sampling from the start and end of each intervention period was performed as described in the Section 3; time points on the axis x are marked as consecutive weeks of the study (weeks 1 and 8 are the start of each intervention period and weeks 4 and 12 are the end of the intervention period). (**A**) MGO levels; (**B**) GO levels; (**C**) 3-DG levels.

Figure 1 illustrates the changes in MGO, GO, and 3-DG levels over the course of the study in subjects randomly assigned to one of two groups in which the intervention sequences were reversed (T-P sequence or P-T sequence). Following CPC treatment, a reduction in MGO concentration was observed (Figure 1A), regardless of the sequence of administration, with MGO levels decreasing from 195.84 nmol/L to 190.97 nmol/L for the T-P sequence and from 187.01 nmol/L to 174.89 nmol/L for the P-T sequence. In subjects who received CPC as the first intervention, a slight increase in GO concentration from 123.26 nmol/L to 127.05 nmol/L was noted. Conversely, for the group receiving CPC as the second intervention, the GO levels decreased from 120.36 nmol/L to 110.26 nmol/L (Figure 1B). Subjects receiving CPC in the T-P sequence had their plasma 3-DG levels slightly increased from 559.44 nmol/L to 561.85 nmol/L, while CPC taken in the second sequence lowered 3-DG levels from 592.96 nmol/L to 567.63 nmol/L (Figure 1C). No significant interaction between treatment and period and no carryover and sequence effect were observed.

The placebo used in this study, maltodextrin, is a low-active polysaccharide derived from the partial hydrolysis of starch [51]. It seemed unlikely that maltodextrin could affect α-DCs levels, particularly since it is commonly used as a food additive, an ingredient in tablet masses, and in human intervention studies as an oral placebo [52]. However, maltodextrin has a high glycemic index, even higher than table sugar, and its consumption causes a sharp postprandial rise in blood glucose levels, potentially resulting in excessive MGO production [6,53]. It is worth noting that, since the study was not designed to measure α-dicarbonyls concentrations, the choice of placebo was based on the primary outcomes of the original study [54].

Previous experimental in vitro studies have found that citrus and pomegranate polyphenols can trap reactive α-dicarbonyls [40,42,44,45]. Under controlled experimental conditions that simulate the physiological environment in terms of pH and temperature, some compounds, such as flavonoids and biguanides, directly react with MGO to form stable adducts, effectively removing the highly reactive MGO from the solution. The adducts resulting from the interaction of MGO with various natural and synthetic compounds have been identified in both in vivo and in vitro experiments, including animal models [55,56] and human subjects [25]. Moreover, it seems that MGO adducts formed by natural compounds like quercetin retain the beneficial physiological effects of the original polyphenol molecule, such as its antioxidant activity [57].

Zhang et al.’s study [55,56] demonstrated that the oral administration of myricetin and taxifolin resulted in the formation of MGO adducts, which were later detected in the urine and feces of mice. When administered to mice via oral gavage, myricetin trapped MGO in the gastrointestinal tract and circulatory system, forming both mono- and di-MGO adducts. Additionally, the phase II metabolites of myricetin, including mono-methylated and di-methylated myricetin, maintained the MGO trapping ability of myricetin, forming their own mono-MGO adducts. This is in line with findings from other studies on genistein, epigallocatechin gallate, and quercetin [58,59,60]. The ability to trap MGO is not exclusive to natural compounds. Kinsky et al.’s research [25] showed that an imidazoline derivative adduct, a result of MGO and metformin interaction, was found in the urine samples of diabetic patients who had been prescribed metformin as part of their regular antidiabetic therapy. However, it is essential to understand that while MGO trapping can occur in both in vitro and in vivo settings, the multifaceted in vivo environment, with its numerous interacting physiological systems and factors, might impact the efficiency and effectiveness of the trapping process compared to controlled in vitro conditions.

The current study used a combination of sweet orange extract and pomegranate concentrate, which together provided 700 mg of the supplement. This supplement contained 450 mg of hesperidin, 60 mg of punicalagin, and 190 mg of other non-characterized components. This complex blend makes it challenging to pinpoint the specific active ingredient responsible for reducing reactive α-DCs. This complex blend makes it challenging to pinpoint the specific active ingredient responsible for reducing reactive α-DCs. There has been a long-standing debate in natural product research over the use of whole plant extracts versus individual plant compounds or phytochemicals for therapeutic applications. Both strategies have their strengths and drawbacks, largely due to the inherent biochemical complexity and the bioavailability of the active ingredients. Whole plant extracts consist of a diverse mixture of phytochemicals, such as phenolic acids, flavonoids, tannins, terpenoids, and others. The holistic use of these extracts is often supported by the ‘entourage effect’ concept, which posits that the combined therapeutic benefits of an entire plant extract exceed that of its isolated components. This is believed to be because of the synergistic interactions between the various compounds [61,62]. Such synergy might arise from the enhanced bioavailability of certain compounds when present alongside other co-existing components [63,64].

However, the complexity of extracts introduces variability in their composition and, thus, their therapeutic efficacy and safety profile. Achieving standardization and maintaining quality control are often challenging [65,66]. On the other hand, isolated plant compounds allow for precise control over dosage and, consequently, therapeutic action. They can be intensively studied, and their pharmacokinetics, pharmacodynamics, and toxicological properties can be thoroughly characterized [67]. Despite these advantages, isolated compounds might lack the synergy found in whole plant extracts, and their bioavailability can be limited [68,69]. To determine the scavenging activity of MGO for specific citrus and pomegranate polyphenols in humans, additional studies with the use of individual compounds are necessary. When considering the use of plant polyphenols or extracts as supplements and nutraceuticals, their bioavailability should always be taken into account. The oral bioavailability of hesperidin, one of two main constituents from the extract combination used in the current study treatment, has also been a topic of intense scrutiny due to its poor absorption characteristics [70]. The poor bioavailability of hesperidin can be primarily attributed to its highly hydrophilic structure and relatively large molecular size (*M*: 610.56 g/mol), which hinders its passive diffusion across the intestinal epithelium. After oral ingestion, hesperidin remains largely unabsorbed in the small intestine and proceeds to the colon, where it undergoes deglycosylation by the local microbiota, being converted into its more lipophilic aglycone form, hesperetin (*M*: 302.27 g/mol) [71]. The released hesperetin can be absorbed and further metabolized in the liver to glucuronide and sulfate conjugates, which have been identified as the main circulating metabolites in plasma [72]. These conjugates are eliminated in the urine within 24 h, and the total urinary recovery of hesperidin metabolites is generally low, indicating a low overall bioavailability [73]. Nevertheless, it is worth noting that MGO trapping activity has been documented for hesperetin. This suggests that even if hesperidin is not absorbed directly into the bloodstream in its original form, its active metabolite might still exert MGO scavenging activity in the systemic circulation. Also, some of the phase II biotransformation metabolites, such as hesperetin-3′-*O*-glucuronide, hesperetin-7-*O*-glucuronide, and hesperetin-3′-*O*-sulfate [74], can also potentially scavenge MGO as their chemical structure meets the basic requirements of being able to bind reactive α-dicarbonyls [75,76]. It is also worth noting that the bioavailability of hesperidin can be affected by several factors, including the food matrix in which it is consumed, individual variations in gut microbiota, and potential interactions with other dietary compounds [77].

The second main ingredient of the tested supplement—punicalagin—is a large molecule (*M*: 1084.71 g/mol) and is classified as an ellagitannin, which makes it less bioavailable due to limited absorption in the gastrointestinal tract. It is known to undergo hydrolysis to smaller polyphenolic compounds, including ellagic acid (EA), and further biotransformation to urolithins and their isomers by gut microbiota, mainly in the colon [78]. Urolithins and isourolithins are the metabolites that appear in plasma and urine, not the initial punicalagin. Therefore, the bioavailability of punicalagin is primarily determined by the bioavailability of the products formed from its hydrolytic degradation [79]. It is important to note that there is considerable inter-individual variability in the production of urolithins/isourolithins, which has been linked to differences in gut microbiota composition among individuals [80]. Urolithin A and urolithin B are the most frequently observed urolithins in human plasma and urine. A recent study by Peng et al. [81] highlighted that urolithin A mitigates AGE formation by trapping reactive MGO, resulting in the creation of mono-MGO-urolithin A adducts.

Taken together, these findings suggest that the active metabolites of the two main components of sweet orange and pomegranate combination potentially have the ability to scavenge MGO by forming adducts with it, and this could be considered as one of the possible mechanisms that led to a significant decrease in the plasma concentration of MGO in study subjects. Unfortunately, participants’ urine samples were not collected, so identifying any potentially formed adducts was not possible. Further in-depth studies are certainly required to determine the mechanism of action of the active constituents found in sweet orange and pomegranate fruit extracts.

Previous clinical studies using the pure flavonoids quercetin-3-glucoside and epicatechin (160 mg/day and 100 mg/day, respectively) have shown that after 4 weeks of oral administration in apparently healthy (pre)hypertensive adults, only quercetin could statistically significantly reduce plasma MGO concentrations by 10.6%. This reduction slightly exceeds the 9.8% result achieved in our study using CPC. Moreover, the study revealed no statistically significant effect of flavonoids on GO and 3-DG plasma concentrations, suggesting that flavonoids may primarily target MGO [82].

The effect size of the reduction in plasma MGO by CPC with 9.8% was also found with pyridoxamine, with a decrease in plasma MGO of 9% [83]. This result was also mirrored in a well-standardized weight loss intervention study with pyridoxamine, which reported a 9% decrease in fasting MGO [84]. These studies were performed in relatively healthy abdominally obese individuals. In our previous research, we showed that MGO concentrations are associated with incident cardiovascular disease in diabetes, with a difference in plasma MGO concentrations of approximately 5% to 13% between diabetic individuals with and without cardiovascular events [85]. Therefore, the 9.8% reduction in plasma MGO, as we found in this study with CPC, could be of clinical relevance.

The reduction in plasma MGO concentration by polyphenols might be a result of actions other than the direct scavenging of MGO. This could be attributed to their strong antioxidant properties [86,87]. Oxidative stress is considered to participate in reductions in the body’s natural detoxification mechanisms such as the expression of Glo1 [88]. The use of substances with strong antioxidant potential such as polyphenols may promote the physiological enhancement of MGO degradation by glo1 [89]. Additionally, the reduction in plasma MGO may also be influenced by the antidiabetic properties of hesperidin or punicalagin, which have shown promise in reducing glucose levels and improving sugar metabolism [90,91,92,93].

The current study was a post hoc analysis of a human randomized cross-over clinical trial. One major limitation is that the trial was not originally designed to identify effects on α-dicarbonyl compounds. Nevertheless, we found a significant reduction in MGO, which aligns with previous in vitro findings. It is crucial to emphasize that our study focused on metabolically healthy elderly individuals with no comorbidities, and the supplementation period itself lasted only 4 weeks. Therefore, the long-term impact of the CPCs might differ in high-risk populations, such as those with diabetes and associated vascular complications.

The identification of methods to reduce MGO concentrations could have substantial implications for preventing and managing MGO stress-related chronic diseases. These interventions may impede or slow down the formation of MGO and MGO-derived AGEs, while also ameliorating oxidative stress and suppressing inflammation. This presents a promising approach to disease management. Therefore, exploring novel therapies aimed at lowering α-DCs, including both pharmacological and non-pharmacological strategies (e.g., dietary modifications and physical exercise), carries significant clinical relevance. Additionally, it is crucial to recognize the potential synergy when these therapies are combined with established treatments, possibly enhancing their effectiveness. Adopting this multifaceted approach could lead to a more comprehensive and effective strategy to address chronic conditions associated with elevated MGO and other α-DCs.

## 3. Materials and Methods

### 3.1. Study Population and Design

The study was conducted from June 2018 to January 2019 and was approved by the local Medical Ethics Committee of the Maastricht University Medical Centre + and performed in accordance with the Declaration of Helsinki of 1975, as amended in 2013, and with the Dutch Regulations on Medical Research involving Human Subjects from 1998. All participants gave written informed consent before data collection. The study was registered at clinicaltrials.gov as NCT03781999. The detailed design of this research containing CONSORT flow diagram of the study participants was previously described by Ahles et al. [54]. In short, 42 elderly, healthy, non-smoking subjects aged 60–75 were recruited through advertisements in the local media. Exclusion criteria included a BMI (in kg/m^2^) lower than 18 and higher than 28, allergy to the investigated product, placebo or citrus fruits, high blood pressure (systolic ≥ 140 mmHg, diastolic ≥ 90 mmHg), abuse of alcohol and drugs, use of beta-blockers, and other medications that may interfere with the study results. Participants were also excluded in case of recent muscle injury less than one month before the start of the study and medical conditions that might influence outcome measure or participant safety during testing, including but not limited to severe cardiovascular disease, cancer, and Parkinson’s disease (concerned measurements of physical activity; not included in this article). This trial was designed as a randomized, placebo-controlled, double-blind, cross-over study. Patients were randomly allocated after enrollment in the study. Randomization was performed using a web service with concealed and random block sizes. Participants ingested a study treatment and placebo capsules for 4 weeks in random order separated by a 4-week period of washout.

### 3.2. Treatment and Placebo

**Figure 2:**
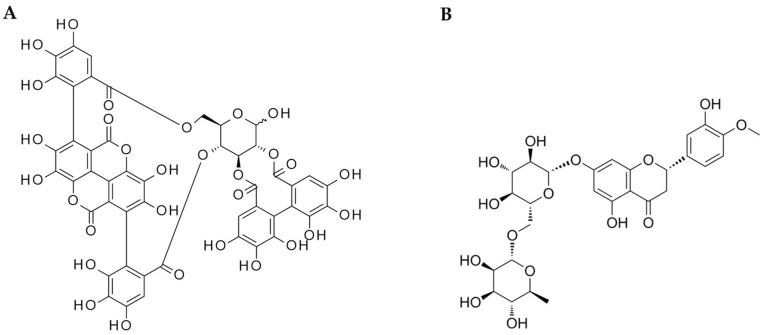
The main components of CPC used as a treatment in the study; (**A**) punicalagin; (**B**) hesperidin.

The treatment was 500 mg *Citrus* × *sinensis* (L.) Osbeck peel extract (containing bioflavonoids) and 200 mg *Punica granatum* L. fruit concentrate (containing ellagitannins and other polyphenols) combination, delivered as a dietary supplement (Citrus & Pomegranate Complex; Actiful^®^, BioActor BV, Maastricht, The Netherlands). The investigational product was standardized chromatographically (LC) by the manufacturer. The daily dose contained 450 mg of hesperidin and 60 mg of punicalagin; the chemical structures of the main treatment components are shown in Figure 2. Maltodextrin (Gonmisol, Barcelona, Spain) was used as a placebo. The study products were formulated into capsules, each of which contained 350 mg of study treatment or placebo. Subjects were asked to ingest 2 gelatin capsules each morning, prior to breakfast with 200 mL of water for 4 weeks. The treatment and placebo were identical in appearance and taste.

### 3.3. Measurement of α-Dicarbonyls in Plasma

Fasting blood samples were collected into Heparin S-Monovette tubes (Sarstedt, Nümbrecht, Germany). Before analysis, all plasma samples stored at −80 °C were thawed and mixed thoroughly. The concentration of α-dicarbonyls in plasma samples was measured with ultra-performance liquid chromatography tandem mass spectrometry (UPLC-MS/MS) according to the method proposed by Scheijen et al. [94] as previously described. In short, 30 μL EDTA plasma samples were mixed with 90 μL *O*-phenylenediamine (10 mg oPD in 10 mL 1.6 M perchloric acid) in an Eppendorf cup. After an overnight (20 h) incubation at room temperature away from light, 20 μL of the internal standard solution (d4-α-DCs) was added. Samples were mixed and subsequently centrifuged for 10 min at 21,000× *g* at a temperature of 4 °C; then, 2.5 μL was injected for UPLC-MS/MS analysis.

### 3.4. Instrumentation

A Waters Acquity I-class system combined with a Xevo TQ-XS mass spectrometer (Waters, Milford, MA, USA) equipped with a reversed-phase C18 column (Acquity UPLC HSS T3, 50 × 2.1 mm, 1.8 μm) was employed for the determination of α-dicarbonyl concentrations.

### 3.5. Statistical Analysis

Statistical analyses were carried out according to a method previously described by Dower et al. [95]. Changes between values of α-dicarbonyls concentration at the start and the end of each 4-week double-blind intervention period were taken as a treatment effect of CPC. All variables were found to be normally distributed. A linear mixed model with compound symmetry as a covariant structure was used to evaluate the treatment effects in this study. The subject was defined as a random effect, while treatment and period were taken as fixed effects. Investigation of treatment–period interaction revealed no carry-over effect of previous treatment and, therefore, it was not included in the final model. Effects of treatment were expressed as mean least squares with 95% Cis, and statistical significance was set at a 2-sided *p* value of 0.05. SPSS Statistics 23 software was used for all analyses. For data visualization, GraphPad Prism 5 software was used.

## 4. Conclusions

In conclusion, this is the first human intervention study to investigate the effects of a combination of sweet orange extract and pomegranate fruit concentrate on reactive α-DCs. This study revealed a significant reduction in plasma MGO concentrations. The MGO scavenging effect exhibited by the active compounds in sweet orange and pomegranate holds promise as a potential therapeutic approach for preventing and managing conditions in which MGO plays a pivotal role.

Based on these initial findings, several recommendations and prospective avenues emerge. It is essential to substantiate these preliminary discoveries with broader studies, involving a more diverse group of participants. Investigating the optimal doses of the combined extracts is crucial for refining the MGO scavenging effect. Moreover, determining the specific molecular pathways through which these compounds act will enhance our understanding and may lead to the development of even more effective interventions. If supported by further research, these findings have the potential to influence the creation of specialized dietary supplements or nutraceuticals targeting MGO-related health concerns. Additionally, this study’s insights hint at the vast potential of phytochemicals in various fruits and plants that might offer therapeutic properties against MGO. Through these revelations, we are establishing a foundation for future research, aiming for innovative solutions to challenges posed by elevated MGO levels.

## Figures and Tables

**Table 1:** Baseline characteristics of 36 healthy subjects randomly allocated at the start of the study.

Characteristic	Minumum	Maximum	Means	SD
Age, y	60	75	66	4
Men:women	-	-	9:27	-
BMI, kg/m^2^	19.7	29.2	25.3	2.1
Plasma α-dicarbonyls, nmol/L				
MGO	145	301	190.1	32.2
GO	53	285	117.7	37.2
3-DG	436	786	569.2	74.6

*n* = 36; BMI, body mass index; MGO, methylglyoxal; GO, glyoxal; 3-DG, 3-deoxyglucosone; SD, standard deviation.

**Table 2:** The total effects of 4-week sweet orange peel extract and pomegranate concentrate combination supplementation on plasma levels of α-dicarbonyls.

Plasma α-Dicarbonyls	Treatment Effect, nmol/L	95% Confidence Interval for Difference		*p*
		Lower Bound	Upper Bound	
MGO	−18.7	−36.7	−0.7	0.042
GO	−7.8	−29.5	13.8	0.473
3-DG	−16.6	−43.9	10.7	0.229

Values are least-square means from a linear mixed model for repeated measures with compound symmetry as the covariant structure, *n* = 36; Treatment effect = (treatment − placebo); *p*, the mean difference was significant at <0.05; Measurements of α-dicarbonyls in fasting plasma samples from the start and end of every intervention period were performed as described in the Section 3.

## Data Availability

The data presented in this study are available on request from the corresponding author.

## Abbreviations

3-DG	3-deoxyglucosone
α-DCs	alpha dicarbonyls
AGEs	advanced glycation endproducts
BMI	body mass index
CEL	*N*^ε^-(1-carboxyethyl)lysine
CPC	citrus and pomegranate extract
Glo1	glyoxalase 1
Glo2	glyoxalase 2
GO	glyoxal
LC	liquid chromatography
MG-H1	*N*^δ^-(5-hydro-5-methyl-4-imidazolone-2-yl)ornithine
MGO	methylglyoxal
oPD	d8-*O*-phenylenediamine
RAGE	receptor for advanced glycation endproducts
RCS	reactive carbonyl species
SD	standard deviation
SEM	standard error of the mean
UHPLC-MS/MS	ultra-high-performance liquid chromatography tandem mass spectrometry
